# Paraoxonase-1 and fetuin-A levels in children with cerebral palsy

**DOI:** 10.55730/1300-0144.5376

**Published:** 2022-03-05

**Authors:** Özlem UNAY DEMİREL, Özlem GÜNGÖR, Seyda İĞNAK TARLIĞ, Meral YÜKSEL

**Affiliations:** 1 Department of Medical Biochemistry and Bahçeşehir University Göztepe Medical Park Hospital Central Laboratory, Faculty of Medicine, Bahçeşehir University, İstanbul, Turkey; 2 Department of Physiotherapy and Rehabilitation, Faculty of Health Sciences, Bahçeşehir University, İstanbul, Turkey; 3 Department of Medical Biology, Faculty of Medicine, Bahçeşehir University, İstanbul, Turkey; 4 Department of Medical Laboratory Techniques, Vocational School of Health Services, Marmara University, İstanbul, Turkey

**Keywords:** Fetuin-A, paraoxanase-1, cerebral palsy, children

## Abstract

**Background/aim:**

Studies mostly focused on risk factors and clinical status in children with cerebral palsy (CP). Various antiinflammatory markers may help us in the early diagnosis and clinical classification of cerebral palsy patients. In this study, the relationship between antiinflammatory marker levels and clinical status in patients with CP is determined. It is the first time that Fetuin-A and Paraoxonase-1 (PON-1) are examined in children with CP.

**Materials and methods:**

The study is conducted on 79 children which are divided into two groups as CP and control. Gross motor function and spasticity are evaluated in addition to biochemical parameters.

**Results:**

There is a statistically significant difference between CP and control group in terms of PON-1 activity, high sensitive C-Reactive Protein, HDL, and total cholesterol levels. There is no statistically significant difference in Fetuin A levels between the two groups.

**Conclusion:**

In suspected CP patients less than 24 months of age who possess prenatal and postnatal risk factors, the determination of PON-1 activity can be considered as a biomarker to support early diagnosis.

## 1. Introduction

Cerebral palsy (CP) is a state of encephalopathy characterized by nonprogressive posture and motor impairment, usually accompanied by speech abnormalities, visual and cognitive defects, and epilepsy resulting from injury to the developing brain that occurs antenatally, perinatally, or early postnatally [1–3]. The incidence of CP is approximately 1.5 to 2.5 per 1000 live births worldwide, whereas in Turkey is 4.4 per 1000 live births [4,5]. Several factors play a role in the etiology of CP which partly explains variations in clinical findings. It may be due to either congenital, genetic, inflammatory, metabolic, anoxic, traumatic, or infectious causes [6]. Hypoxic-ischemic and inflammatory conditions are among the key factors which lead to cell death and cell process loss in CP patients [3]. An antioxidant and antiinflammatory enzyme known as Paraoxonase 1 (PON-1) a member of the paraoxonase family which is primarily expressed in the liver, is transported in the circulation via high-density lipoprotein (HDL). It is well known that PON-1 prevents further damage with its peroxidase and lactonase activity during the inflammatory process and in this way provides antioxidant and antiinflammatory actions. Changes in circulating PON-1 concentrations are reported in certain diseases like cardiovascular and neurological diseases in which oxidative stress is involved [7,8]. Another antiinflammatory marker, Fetuin-A has a glycoprotein structure and it belongs to the cystatin protein family which is mainly synthesized in the liver. Fetuin-A shows neuroprotective effect by its antiinflammatory effect in cerebral ischemia. It is known that Fetuin-A plays a role in the remodeling of the neonatal brain and it is reactivated in the damaged brain [9,10]. Therefore, increased Fetuin-A level may reduce the inflammatory response and prevent further brain damage [11]. Cerebral palsy patients can be clinically assessed by the gross motor function classification system (GMFCS) which is the gold standard for evaluating motor function in CP patients [12]. Moreover, fine motor function test and modified Ashworth scale are also generally used in this group to determine the degree of spasticity [13].

To date, studies have focused on risk factors and clinical status in children with CP. According to our knowledge, there is no study evaluating antiinflammatory markers like PON-1 activity and Fetuin-A levels and clinical status. In our study, we aimed to investigate the relationship between antiinflammatory markers such as PON-1, Fetuin- A, high sensitive C-Reactive Protein (hsCRP) as well as blood lipid parameters and clinical status in patients with CP.

## 2. Materials and methods

### 2.1. Study design

The patients with cerebral palsy were admitted to the physical therapy and rehabilitation unit in the Göztepe Medical Park Hospital. The study was conducted with 79 children which were divided into two groups as CP (n = 38) and control (n = 41). The patient’s history was taken and a physical examination was performed. The control group was selected from healthy children outpatient clinic. Age, sex, and other demographic characteristics of the control group were matched accordingly with the CP group. Children with epilepsy, head trauma, any kind of neuropsychiatric disorder and metabolic disorder, acute or chronic infection were excluded from the control group. The study protocol was approved by the local ethics committee and written informed consent for the study was obtained from the legal guardian of all subjects.

### 2.2. Assessment of gross motor function and spasticity

The Modified Ashworth scale (MAS) was used to determine spasticity. The total score was obtained from the addition of individual scores of hip, knee, and ankle for both lower limbs. Gross motor functional ability was evaluated by the GMFM-66 [14,15].

### 2.3. Biochemical analysis

After overnight fasting, blood was collected. Serum was separated using centrifugation at 1500 × g for ten min. Serum samples were aliquoted and stored at −80 °C until analysis. High sensitivity CRP (hsCRP), total cholesterol, LDL (low-density lipoprotein), triglyceride, and HDL (high-density lipoprotein) levels were determined by Abbott Architect ci8200 autoanalyzer (Abbott Park, IL, USA). Paraoxonase activity was measured by the initial rate of paraoxon hydrolysis to yield pnitrophenol at 25 °C with spectrophotometric technique which was performed at 405 nm [16]. Fetuin-A level was evaluated by using enzyme-linked immunosorbent assay (ELISA) Aviscera Bioscience (Santa Clara, California, USA) according to the manufacturer’s instructions. Intra- and inter assay coefficients of variation were found to be 4.5%.

### 2.4. Statistical analysis

In this study, IBM SPSS Statistics 22 for statistical analysis (SPSS IBM, Turkey) programs were used. The suitability of the variables to normal distribution was evaluated by Shapiro Wilks test, Q-Q graphs, and histograms. Descriptive statistical methods (mean, standard deviation, frequency, percentage, median, quartiles) and also quantitative data that did not show normal distribution were used for the Mann-Whitney U test. Pearson chi-square test and Continuity (Yates) corrected chi-square tests were used to evaluate the qualitative data. Significance was accepted as p < 0.05.

## 3. Results

We included 79 children and 38 of them were CP whereas 41 were in the control group in our study. The general characteristics of study groups are shown in Table 1. The clinical findings of CP group were evaluated by physiatrists. Moreover, physical examination findings of children in the CP group in terms of gross motor function, fine motor function, modified Ashworth scale, and reflex grades are represented in Table 2. Most of the patients had GMF of Grade 3–5. The assessment of fine motor function of our study group was mostly Grade 1. Since the groups based on grades were not homogenously distributed, we did not perform statistical analysis. When we evaluated the biochemical parameters we found a statistically significant difference between the CP and the control group in terms of PON-1 activity, hsCRP, HDL, and total cholesterol levels. Biochemical test results are presented in Table 3. There was no statistically significant difference in Fetuin-A levels between the two groups. However Fetuin-A levels were significantly higher in CP children without maternal intrauterine infection (p = 0.043, p < 0.05), in children without congenital brain malformations (p = 0.012, p < 0.05), in patients without bradycardia and hypoxia in perinatal period (p = 0.036, p < 0.05), in patients without mental retardation (p = 0.020, p < 0.05), in patients without salivation (p = 0.012, p < 0.05), in patients without speech problems (p = 0.032, p < 0.05), in patients without musculoskeletal disorders (p = 0.002, p < 0.05) when compared to CP children with these problems. On the other hand, Fetuin-A, LDL, and total cholesterol levels were significantly higher in children with CP who were not affected by socio-economic factors than those affected (p < 0.05). The distribution of risk factors, perinatal and postnatal characteristics are summarized respectively in Table 4–5.

## 4. Discussion

This study shows that PON-1 activity is significantly lower and hsCRP levels are higher in children with CP when compared with the control group. PON-1 which is incorporated into HDL structure prevents lipoprotein oxidation via lipid peroxide hydrolysis in oxidized LDL and HDL molecules [8,17]. It is also known that low PON-1 activity is a risk factor for the development of cardiovascular disease independently of HDL level [8,18]. We detected low PON-1 activity and increased HDL level in children with cerebral palsy in our study group, which may be a risk factor in advancing age. Therefore, determination of PON-1 activity may play a role in the early diagnosis of cerebral palsy and follow-up in long term may give information about the tendency of cardiovascular diseases. There are some studies done for the determination of PON-1 activity in the pediatric population [19–21]. It is shown that low levels of PON-1 enzyme are maintained between 2 to 7 years of age [22–24]. In our study, most of the children in both the CP and the control group were older than 2 years of age.

Another routine laboratory test which is hsCRP is regarded as a prognostic marker in cerebrovascular and cardiovascular diseases in addition to its inflammatory action [25–27]. There was a statistically significant increase in hsCRP levels in CP when compared to the control group. Pingel et al. found that CRP levels are 8 times higher in children with CP when compared to healthy adults. Furthermore, CRP indicates catabolism of muscle tissue and it might play a role between poor muscle function and systemic inflammation [28]. Moreover, Fetuin-A which plays a role in the remodeling of newborn brain tissue is reactivated in damaged brain tissue such as ischemia and inflammation [9]. However, we did not find any significant difference when we compared the two groups. According to the studies, there is no definitive reference range of Fetuin-A in infants and children. This may be partly explained by differences in diet, physical activity, and genetics [11]. As far as we know antiinflammatory markers such as PON-1 and Fetuin-A levels are not determined in children with CP.

Although many risk factors of CP were identified up to now, half of the diagnosed patients did not have any risk factors [29]. In our study, inherited diseases and socioeconomic factors are encountered approximately in one-third of the CP patients. We found increased levels of Fetuin-A, LDL, and total cholesterol in CP patients who were not affected by socioeconomic factors.

Besides the antiinflammatory markers, a blood lipid profile was also determined. In some studies, it was indicated that CP patients have more tendency towards vascular diseases which can be explained by increased inflammatory status and impaired blood lipid profile [30–31]. We observed a significant difference in HDL and total cholesterol levels whereas there was no difference in LDL cholesterol levels between the two groups. Cece et al. reported that there was no difference by means of total cholesterol, LDL, HDL, and triglyceride levels in children with CP and the control group [32]. Another study conducted by McPhee P et al. found that LDL levels were high in adult CP patients in relation to reference range [33]. Specific biomarkers can be used to evaluate the clinical course in CP patients. These patients are characterized by observations of caregivers such as head lag, inability to grasp, not sitting, etc. The diagnosis of cerebral palsy is made by evaluating the neurological findings along with the associated risk factors. Due to the variations in clinical indicators, diagnosis of CP is often delayed until 12–24 months of age. It is known that earlier diagnosis of CP provides an opportunity for initiating therapies in a period when brain development is rapid and neuroplasticity occurs [29,34].

GMFCS not only gives information about the motor function of the patient at the present time but also predicts long-term physical limitations considering that the patient may need physical assistance or mobility equipment in the future. GMFCS is a 5 level clinical classification system and it is not an applicable test for children younger than 24 months [29,34]. In our study, we had only 3 patients younger than 24 months of age. Noble et al. used a modified Ashworth scale to evaluate the degree of spasticity which can also explain the correlation between spasticity and gross motor function suggested by other studies [35]. In our study, 51.2% of patients were grade 4 in GMFCS and 47.4% of patients were grade 2 in the modified Ashworth scale. Because of the uneven distribution of patients correlation between laboratory parameters and clinical status could not be performed.

There are several limitations to our study. First of all, we could not find any relation between laboratory parameters and GMFCS, modified Ashworth scale because of the small sample size and uneven distribution of groups. Since Fetuin-A, is increased in CP patients, there was no statistically significant difference so it would be appropriate to investigate in more detail with increased sample size by stabilizing biological variables such as diet and exercise. Furthermore, studies can be focused on the role of PON-1 in CP patients for predicting cardiovascular and cerebrovascular events which may be observed later in life span.

In suspected CP patients less than 24 months of age who possess prenatal and postnatal risk factors, the determination of PON-1 activity can be considered as a biomarker to support early diagnosis.

## Figures and Tables

**Table 1:** Evaluation of general characteristics of children by groups.

		CP group (n = 38)	Control group (n = 41)	Total (n = 79)
**Age (months)**	**Mean ± SD**	84.2 ± 40.2	67.8 ± 61.5	75.7 ± 52.7
**Sex**	**Female**	13 (34.2%)	15 (36.6%)	28 (35.4%)
	**Male**	25 (65.8%)	26 (63.4%)	51 (64.6%)
**Age group**	**≤24 months**	3 (8%)	5 (12.2%)	8 (10.1%)
	**25–72 months**	14 (36.8%)	12 (29.3%)	26 (33%)
	**73–108 months**	14 (36.8%)	11 (26.8%)	25 (31.6%)
	**≥109 months**	7 (18.4%)	13 (31.7%)	20 (25.3%)

Min-Max: Minimum-Maximum, Mean ± SD: Mean ± Standard Deviation

**Table 2:** Distribution of physical examination results of children in CP group (n = 38).

	Grade	n (%)
**Gross motor function**	0	1 (2.6%)
	1	3 (7.7%)
	2	4 (10.3%)
	3	7 (17.9 %)
	4	20 (51.2%)
	5	4 (10.3%)
**Fine motor function**	0	6 (15.8 %)
	1	31 (81.6 %)
	2	1 (2.6%)
**Ashworth scale (modified)**	0	5 (13.1 %)
	1	9 (23.7%)
	2	18 (47.4%)
	3	6 (15.8%)
**Reflexes**	0	11 (29 %)
	1	7 (18.4%)
	2	19 (50%)
	3	1 (2.6%)

**Table 3:** Evaluation of biochemical parameters of children by groups.

Biochemical parameters	CP group (n = 38) Median (min–max)	Control group (n = 41) Median (min–max)	p-value
**PON1 (U/L)**	102.20 (96–121.8)	133.4 (104.9–156.4)	**p ** ***<*** ** 0.001** *
**Fetui-A (pg/mL)**	984 (368–1258)	421 (278–649)	p = 0.082
**HDL (mg/dL)**	50 (31–68)	32 (9–76)	**p = 0.002** *
**LDL (mg/dL)**	88 (57–147)	79 (32–165)	p = 0.060
**Total cholesterol (mg/dL)**	165.5 (119–219)	127 (79–282)	**p = 0.002** *
**hsCRP (mg/L)**	0.48 (0.2–12.69)	1.12 (0.2–4.57)	p *=* 0.052
**Triglyceride (mg/dL)**	106 (54–270)	110 (36–502)	p = 0.851

Mann Whitney U Test

* p < 0.05

**Table 4:** Distribution of risk factors for children in the CP group (n = 38).

CP Risk Factors	Yes n (%)
Inherited diseases	12 (31.6)
Maternal intrauterine infections	8 (21.1)
Metabolic diseases of the mother	5 (13.2)
Intrauterine anoxia or decreased blood flow to the fetus	7 (18.4)
Rh mismatch-development of kernicterus as a result of AB0 and Rh mismatch	3 (7.9)
Exposure to radiation. teratogens in the first trimester	1 (2.6)
Chemical poisoning	2 (5.3)
Complicated pregnancy. bleeding	4 (10.5)
Congenital brain malformations	5 (13.2)
Socioeconomic factors	12 (31.6)
Reproductive insufficiency	3 (7.9)
Maternal mental retardations. convulsions	-
Multiple pregnancy	10 (26.3)
Abortion trials	10 (26.3)
Fetus posture disorders due to lack of amniotic fluid	2 (5.3)
Abdominal trauma	1 (2.6)
Prenatal cerebral hemorrhage	-

**Table 5:** Distribution of perinatal and postnatal characteristics in CP group (n = 38).

	Yes n (%)
**CP perinatal period**	
Premature < 36 weeks	18 (47.4)
Low birth weight (< 2500 g)	20 (52.6)
Difficult/intervention delivery	14 (36.8)
Abnormal arrival	2 (5.3)
Intracranial hemorrhage	1 (2.6)
Trauma	1 (2.6)
Infection	9 (23.7)
Bradycardia and hypoxia	11 (28.9)
Low APGAR score	11 (28.9)
Anoxia	10 (26.3)
**CP postnatal period**	
Trauma	2 (5.3)
Infection	7 (18.4)
Intracranial hemorrhage	1 (2.6)
Coagulopathies	-
Convulsion	5 (13.2)
Hyperbilirubinemia	11 (28.9)
Arteriovenous malformations	-
Anoxia	1 (2.6)
Inflammatory-immunological causes	-
Intracranial pathologies	1 (2.6)
